# Organoid modeling for cancer precision medicine

**DOI:** 10.1186/s13073-015-0158-y

**Published:** 2015-03-31

**Authors:** Michael A Cantrell, Calvin J Kuo

**Affiliations:** Department of Medicine, Division of Hematology, Stanford University School of Medicine, Stanford, CA 94305 USA

## Abstract

Three-dimensional organotypic culture models show great promise as a tool for cancer precision medicine, with potential applications for oncogene modeling, gene discovery and chemosensitivity studies.

## Cancer precision medicine

Recent advances in next-generation sequencing have afforded insight into the genomic complexity of cancer, revealing mutational and epigenetic events, as well as chromosomal instability. The daunting magnitude of tumor heterogeneity is now clear, both within an individual’s primary tumor or metastases, and between patients. The concept of cancer precision medicine has arisen from these analyses; the idea being that treatments should be tailored to a tumor’s genetic composition. Notable successes with mutation-driven targeted therapies have already been achieved.

Large-scale projects such as The Cancer Genome Atlas (TCGA) have sought to capitalize on the promise of precision medicine, combining integrative sequencing, proteomics, tissue banking and data analysis centers with public data distribution. Although cancer study cohorts are not yet sufficiently powered for full detection of rare tumor subtypes or rare ‘long-tail’ genomic events, and few analytical data have been validated, the promise of this approach has been recently promulgated in the US Government Precision Medicine Initiative. Under this initiative, large cohorts of up to one-million participants will be assembled, with goals including the identification of genomic drivers in cancer and the derivation of new personalized treatments.

To fully realize the potential of cancer precision medicine, bioinformatic annotation of the vast troves of available genetic and epigenetic data is underway via cancer-specific and pan-cancer analyses. It is essential that candidate loci, however promising they appear according to bioinformatic criteria, be confirmed as *bona fide* ‘driver’ oncogenes by systematic functional validation; these targets can then be prioritized for therapeutic development. Such validation can be achieved by two complementary strategies: either a ‘bottom-up’ approach in which candidate oncogenic mutations are systematically engineered into normal wild-type tissue, or a ‘top-down’ approach in which tumor tissue is exposed to genetically manipulated oncogene candidates or therapeutics. In both cases, the effects of perturbations on tumorigenicity can be systematically assessed.

Functional validation through contemporary screening methods may be carried out either *in vitro* or *in vivo*. Traditional *in vitro* cancer assays use transformed cell lines that are easily propagated, typically in a two-dimensional monolayer. These are experimentally tractable by diverse means, such as viral transduction, genome engineering, pharmacologic treatment, and multiplexed screening geometries. However, such cell lines are far from wild type, and their extensive culture has often engendered highly complex genetic backgrounds that complicate interpretation - especially when considering genes with incremental effects on tumor progression. On the other hand, *in vivo* mouse ‘avatars’ (patient-derived xenografts) or genetically engineered mouse models (GEMMs) allow three-dimensional tissue context and stromal recapitulation, but have the potential disadvantages of non-human context, low throughput and high cost. A recently developed alternative, the three-dimensional culture of organoids, shows promise for both bottom-up and top-down screening.

## Bottom-up cancer modeling in wild-type tissue organoids

Organoids are miniature forms of tissues that exhibit endogenous three-dimensional organ architecture, multilineage differentiation, and include stem cells in a simple *in vitro* system. Human and mouse organoids composed exclusively of epithelium can be propagated from previously intractable normal gastrointestinal tissues such as intestine, pancreas, stomach and liver, typically in a submerged extracellular matrix geometry using defined growth factors to supplement stromal or niche signals [1]. In addition, murine organoids containing both epithelium and mesenchyme have been propagated in a non-submerged air-liquid interface (ALI) that does not require growth factor supplementation [2]. Furthermore, human induced pluripotent stem cells can be differentiated to intestinal fates and expanded as epithelial/mesenchymal organoids [3], and ‘conditionally reprogrammed’ primary human cells on stemness-promoting fibroblast feeder layers can be grown in a two-dimensional monolayer or in a three-dimensional ALI [4].

Such wild-type gastrointestinal organoids represent an opportunity for bottom-up functional oncogene validation; candidate oncogenic mutations can be introduced into wild-type tissue with a clean genetic background, either singly or in combination. Tumor-suppressive loci such as *Trp53*, *Pten* and *Apc* have been knocked down singly in mouse small intestinal organoids [5]. We successfully obtained *in vitro* oncogenic transformation of murine ALI organoids from colon, stomach and pancreas to adenocarcinoma by the combinatorial introduction of *Apc*, *Kras*^*G12D*^, *Trp53* and *Smad4* mutations [2]. In these studies, mutations were introduced by a combination of floxed alleles from GEMM models and superimposed lentiviral cDNA/short hairpin RNA (shRNA) transduction; the resultant organoids displayed foci of high-grade dysplasia *in vitro*, and tumorigenicity with varying degrees of adenocarcinoma histology upon *in vivo* subcutaneous transplantation. Recently, mouse pancreatic organoids from a lox-stop-lox *Kras*^*G12D*^ GEMM model were grown in submerged extracellular matrix culture. The resultant *Kras*^*G12D*^ organoids produced pre-invasive, pancreatic intraepithelial neoplasia-like tumors 2 weeks after orthotopic transplantation, whereas the addition of superimposed p53 shRNA knockdown yielded invasive carcinoma by 3 months [6].

The modeling of cancer mutations in human organoids represents a promising new experimental horizon. Human colon organoids have recently been manipulated by clustered regularly interspaced short palindromic repeats (CRISPR)/Cas9 gene editing methods to engineer deletions of *APC*, *TP53* and *SMAD4*, combined with point mutations in *KRAS*^*G12V*^ and *PIK3CA*^*E545K*^ [7]. By manipulating the various components of the basal organoid media in the context of gene editing, tailored selection for the engineered mutations was accomplished. Interestingly, even with five engineered driver mutations, cultured organoids had no morphological phenotype and were genomically stable. Xenografted organoids from the submerged culture system did not metastasize when transduced with the five-gene module; however, metastases were produced when mutations were introduced into a colorectal adenoma line with proven chromosomal instability. This result suggests that other genetic or epigenetic events, even those thought to be ‘passenger’ mutations, may be necessary to evoke the full range of tumorigenic phenotypes in organoids. Although novel oncogene validation has not been performed in human gastrointestinal organoids, murine organoids have been used to confirm the microRNA *miR-483* as a driver oncogene from the TCGA colorectal cancer 11p15.5 amplicon [2]. Similarly, knockdown of the *Tgfbr2* gene, encoding the TGF-β receptor 2, in murine *Cdh1*^*−/−*^; *Trp53*^*−/−*^ gastric organoids confirmed tumor suppressor activity with increased metastasis upon *in vivo* transplantation [8], and gastric cancer *RhoA* mutations have been modeled in intestinal organoids [9].

These studies raise several issues for future investigation. Depending on the organoid system employed, common driver mutations may not be sufficient to induce *in vitro* dysplasia, *in vivo* tumorigenicity or metastasis. Numerous variables exist between different methods, including the nature of growth factor supplementation, culture geometry, extracellular matrix type, inclusion or exclusion of stroma, and the use of mouse versus human tissue - all of which could influence transformation susceptibility. Issues of potential genetic drift and accumulation of secondary passenger mutations may well be no different for organoids than for transformed cell lines, and these additional genetic events could be required for manifestation of the fully transformed phenotype. Heterogeneity within oncogene-engineered organoids may arise, as is well documented within human tumors, and may be further compounded by baseline genetic variation between humans from which different wild-type organoids are derived. Furthermore, optimal oncogene validation may require contextual modeling within a background of known and associated drivers that are co-mutated in human cancer. Nevertheless, such organoids represent an extremely promising approach for bottom-up oncogene modeling and discovery.

## Top-down target validation and precision medicine

Tumor organoids offer numerous prospects for top-down precision medicine applications. Recent studies have demonstrated the feasibility of three-dimensional organoid culture of primary human pancreatic and colon tumors that display *in vivo* tumorigenicity upon transplantation [6,7]. Patient-derived tumor organoids can be subjected to genetic manipulation to elucidate the causes of specific cancer phenotypes. By extension, *in vitro* tumor organoid models could potentially be used to assess sensitivity to diverse classes of therapeutics, with the attendant promise of patient response prediction, similar to mouse avatar approaches. A patient’s tumor organoids could be exposed to an empirically chosen panel of therapeutics, and the results could be used to predict tumor response within an actionable timeframe. Analogous predictive studies using conditionally reprogrammed lung cancer cells in two-dimensional culture have been recently described [10]. Furthermore, compound screening in organoids carrying *de novo* engineered mutations holds promise for the development of targeted therapies against rigorously characterized mutational backgrounds.

Numerous questions remain for top-down models, including whether three-dimensional methods offer advantages over two-dimensional methods for gene discovery and drug screening, and whether *in vitro* organoid therapeutic predictions correlate with actual patient outcomes. Certainly, sampling bias of the initial tumor, culture medium and culture geometries could potentially modulate drug effects. On the one hand, the results of drug predictive assays, whether based on organoids, avatars or other methods, can be obtained completely independently of tumor sequencing. On the other hand, organoid therapeutic sensitivity data could be complementary to tumor genetic testing, providing actionable data that would suggest treatment options beyond those provided by sequencing-based approaches. Furthermore, correlations may be drawn between organoid sensitivity and genomic sequencing data. Such correlations would give physicians the power to provide evidence-based care to patients with tumor types for which few treatment options exist. Finally, these organoid-based strategies could be generalized to model the biology and treatment responses of any number of non-malignant disorders, exploiting the ability to culture primary human cells in a physiologic manner.

## Abbreviations

ALI: Air-liquid interface
CRISPR: Clustered regularly interspaced short palindromic repeats
GEMM: Genetically engineered mouse model
shRNA: Short hairpin RNA
TCGA: The Cancer Genome Atlas
